# Kinetic Analysis for Macrocyclizations Involving Anionic Template at the Transition State

**DOI:** 10.1100/2012/748251

**Published:** 2012-04-22

**Authors:** Vicente Martí-Centelles, M. Isabel Burguete, Santiago V. Luis

**Affiliations:** Departamento de Química Inorgánica y Orgánica, Universitat Jaume I, E12071 Castellón de la Plana, Spain

## Abstract

Several kinetic models for the macrocyclization of a C_2_ pseudopeptide with a dihalide through a S_N_2 reaction have been developed. These models not only focus on the kinetic analysis of the main macrocyclization reaction, but also consider the competitive oligomerization/polymerization processes yielding undesired oligomeric/polymeric byproducts. The effect of anions has also been included in the kinetic models, as they can act as catalytic templates in the transition state reducing and stabilizing the transition state. The corresponding differential equation systems for each kinetic model can be solved numerically. Through a comprehensive analysis of these results, it is possible to obtain a better understanding of the different parameters that are involved in the macrocyclization reaction mechanism and to develop strategies for the optimization of the desired processes.

## 1. Introduction

**Scheme 1:**
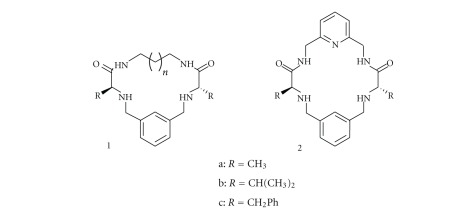
Macrocycles synthesized [4, 8].

All chemical phenomena involve the modification of chemical interactions. Covalent bonds are critical for the building of organic molecules and for their formation thermodynamic, and kinetic factors need to be considered. On the other hand, noncovalent interactions, like hydrogen bonding or van der Waals forces, are essential to understand the behaviour of biological systems, including the formation of covalent bonds, and this is at the base of the strong development of the area of supramolecular chemistry [1]. In this context, pseudopeptidic compounds are good receptors of anions due to the coordination properties of the amide groups [2, 3]. For this purpose, the preorganization provided by macrocyclic structures is important, and the preparation of such structures from C_2_ pseudopeptidic bis(amidoamines) has been investigated in detail in our research group [4–7]. Previous studies focused on compounds such as 1 (Scheme 1) [4]. In order to introduce further functionalities in the system, we considered the preparation of macrocycles containing a pyridine fragment like 2. Initial experiments for the preparation of 2a, 2b, and 2c revealed soon that the corresponding macrocyclization is more efficient than that observed for 1, not only in terms of the yields obtained but also in terms of its kinetics [8]. Besides, a clear catalytic effect was observed for the presence of some anions, including bromide and chloride. In order to analyse in detail this phenomenon, the corresponding kinetic study requires being able to consider the viability of the different kinetic models that can be associated to the system under consideration. 

Independently of the kinetic model considered, the formation of macrocycles 1 and 2, according to the synthetic procedure developed, must take place in two steps. In the first reaction step, one of the C–N bonds is formed through an intermolecular S_N_2 reaction. This is accompanied by the formation of one equivalent of acid, which must be neutralized by a base present in the reaction medium. In the second reaction step, also known as the macrocyclization reaction step, the second C–N bond is formed yielding also one equivalent of acid. This is usually the most critical step for obtaining an efficient macrocyclization. Entropic and enthalpic factors play an important role in the preparation of macrocyclic structures [9–11]. To favour the macrocyclization over the competing oligomerization/polymerization reactions, the open-chain precursor must be preorganized in a folded conformation, therefore approaching both reacting ends of the open-chain precursor [12–17]. In this regard, anions can act as an external stimulus. The binding of the anion to two sites of the open-chain precursor can change the preferred conformation to a folded one [18–20]. 

**Scheme 2:**
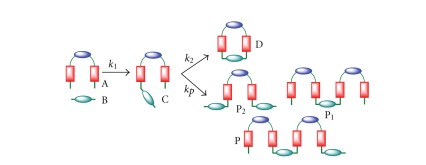
General representation of the different competing processes in the macrocyclization under study.

 In the case of polymerization, development of appropriate kinetic models has allowed to predict the final distribution of polymers according to the initial polymerization conditions [21]. In the case of macrocyclizations, the situation is even more complex (Scheme 2), and the development of appropriate kinetic models is of key importance to understand and optimize the macrocyclization reactions and to provide strategies to control the most important side-reactions.

 In order to obtain workable kinetic models, we must assume that the different kinetic constants for the nucleophilic substitution reactions giving place to open-chain compounds are essentially identical (*k*_1_ = *k*_*p*_). This assumption is similar to those considered for other multistep-processes, for instance, propagation steps in polymerization processes or nanoparticles growing [21–44]. This allows obtaining kinetic models to which the experimental kinetic data can be fitted. An analysis of the relative importance of the different parameters and processes considered is possible after this treatment [8]. Here, a comprehensive study of the different possible kinetic models for the macrocyclization reaction, taking into account the main process and the undesirable side reactions, is detailed.

## 2. Results and Discussion

 A broad variety of kinetic models for the macrocyclization reaction can be developed. Each reaction mechanism takes into account, in a different way, the possible side reactions competing with the main macrocyclization reaction. Therefore, all possible variables that affect to the system can be evaluated and their effect over the macrocyclization yield analysed in detail. For the development of the different models, it has been always considered that the first reaction step is faster than the macrocyclization reaction step. This is reasonable taking into account the characteristics of both processes and the fact that the macrocyclization step (*k*_2_) involves a transition state with a less favourable folded conformation. This is in good agreement with experimental data for this kind of processes and is also predicted from high-level theoretical calculations [8]. On the other hand, when appropriate, it has been assumed that *k*_1_ = *k*_*p*_ according to the reasons considered above.

### 2.1. Kinetic Model 1

**Scheme 3:**
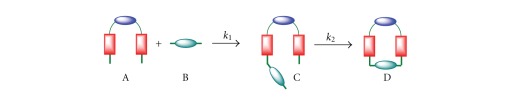
Reaction scheme for kinetic model 1.

 The simplest kinetic model for the macrocyclization reaction is the one considering that no side reactions take place (Scheme 3). Only the two steps leading to the formation of the macrocyclic product are considered

 In a more compact style, reactions of Scheme 3 can be represented as in (1)

(1) $$\begin{array}{c} \text{A}+\text{B}\overset{k_{1}}{\to }\text{C}\\ \text{C}\overset{k_{2}}{\to }\text{D}. \end{array}$$

In (1), A is the bis(amidoamine), B is the dihalide, C is the reaction intermediate, and D is the macrocyclic product. Assuming that all the reactions are irreversible, the following simple differential equations are obtained. The starting materials A and B disappear by a second-order rate reaction (2) and (3)

(2) $$\frac{d\left[\text{A}\right]}{dt}=-k_{1}\left[\text{A}\right]\left[\text{B}\right]$$

(3) $$\frac{d\left[\text{B}\right]}{dt}=-k_{1}\left[\text{A}\right]\left[\text{B}\right].$$

The reaction intermediate should be formed through a second-order rate reaction and disappears by a first-order rate reaction (4)

(4) $$\frac{d\left[\text{C}\right]}{dt}=k_{1}\left[\text{A}\right]\left[\text{B}\right]-k_{2}\left[\text{C}\right].$$

**Figure 1:**
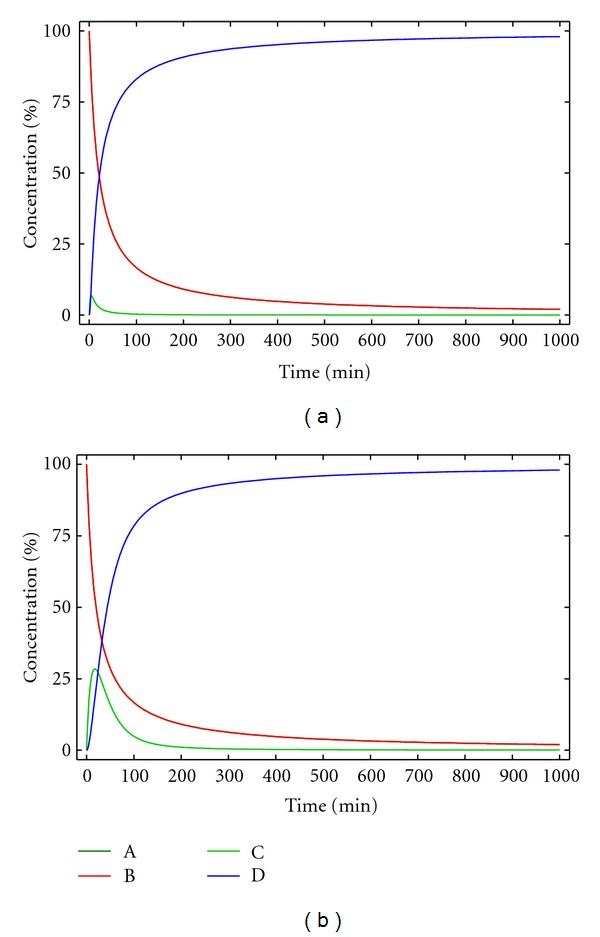
Concentration of the reactants, products, and reaction intermediate versus time for kinetic model 1. [A]_0_ = [B]_0_ = 0.005 M, *k*_1_ = 10 M^−1^ min^−1^ and (a) *k*_2_ = 0.5 min^−1^, (b) *k*_2_ = 0.05 min^−1^.

The macrocyclic product D is formed by a first-order rate reaction

(5) $$\frac{d\left[\text{D}\right]}{dt}=k_{2}\left[\text{C}\right],$$

where [A], [B], [C], and [D] are the molar concentrations at time *t* of compounds A, B, C, and D. The solution of differential equations (2)–(5) can be obtained numerically with the *NDSolve* command implemented in Mathematica [45]. Knowing the values of the initial concentration of reactants ([A]_0_, [B]_0_), the concentration of each chemical specie is an *interpolating function* that can be plotted, integrated, differentiated, or fitted to experimental data according to the values of *k*_1_ and *k*_2_.  Figure 1 shows the representation of the solutions of the system of differential equations for typical values of *k*_1_, *k*_2_ and [A]_0_, [B]_0_ for the kinetic model 1.

According to this kinetic model, the yield of the macrocyclic compound should be 100% because there are no side reactions, and therefore no byproducts are formed. Comparison of Figures 1(a) and 1(b), allows to observe that the smaller *k*_2_ the bigger the concentration of the reaction intermediate C at the beginning of the reaction. According to the experimental results, this is not a valid model as the yield never reaches 100%, and the presence of different side products can be identified [4, 8].

### 2.2. Kinetic Model 2

 This kinetic model assumes that the reaction is performed in the absence of any base added and allows to understand its critical role in the reaction. As far as bis(amidoamine) A is more basic than the macrocyclic compound D, the HBr formed will protonate bis(amidoamine) A. It can be experimentally observed that compound A—in the case of the synthesis of macrocycles 2—precipitates when triprotonated with 3 HBr molecules. Therefore, the concentration of A is reduced (1/3 of the HBr formed), and this needs to be taken into account

(6) $$\begin{array}{c} \text{A}+\text{B}\overset{k_{1}}{\to }\text{C}+\text{HBr}\\ \text{C}\overset{k_{2}}{\to }\text{D}+\text{HBr}\\ \text{A}+3\text{HBr}\to \text{A}{\text{H}}_{3}\text{B}{\text{r}}_{3}\left(\text{s}\right). \end{array}$$

The system of differential equations for the disappearance of reactants is described in (7)

(7) $$\begin{array}{c} \frac{d\left[\text{A}\right]}{dt}=-k_{1}\left[\text{A}\right]\left[\text{B}\right]-\frac{1}{3}\frac{d\left[\text{HBr}\right]}{dt}\\ \frac{d\left[\text{B}\right]}{dt}=-k_{1}\left[\text{A}\right]\left[\text{B}\right]. \end{array}$$

The reaction intermediate is formed by a second-order rate reaction and disappears by a first-order rate reaction, (8)

(8) $$\frac{d\left[\text{C}\right]}{dt}=k_{1}\left[\text{A}\right]\left[\text{B}\right]-k_{2}\left[\text{C}\right].$$

And the macrocyclic product D is formed by a first-order rate reaction

(9) $$\frac{d\left[\text{D}\right]}{dt}=k_{2}\left[\text{C}\right].$$

HBr is formed in the first reaction step and in the macrocyclization reaction step according to (10)

(10) $$\frac{d\left[\text{HBr}\right]}{dt}=k_{1}\left[\text{A}\right]\left[\text{B}\right]+k_{2}\left[\text{C}\right],$$

where [HBr] is the molar concentration at time *t* of HBr. It has to be taken into account, however, that HBr will be intermediately and quantitatively transformed into the corresponding salt. This, however, has not been considered in (10) as it does not affect to the overall kinetics of the process.

**Figure 2:**
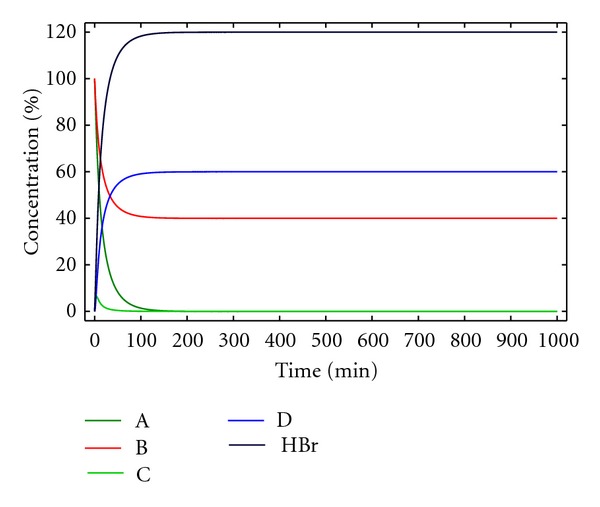
Concentration of the reactants, products, and reaction intermediates versus time for kinetic model 2. [A]_0_ = [B]_0_ = 0.005 M, *k*_1_ = 10 M^−1^ min^−1^ and *k*_2_ = 0.5 min^−1^.

 A solution of this set of differential equations, using the same approach as for model 1, is displayed in Figure 2.

In this case, the yield for the formation of macrocycle D is 60%, while 40% of B has not reacted, for a 1 : 1 A/B initial stoichiometry This agrees well with the decreased yields observed experimentally in the absence of base. The overall process can be depicted according to (11)

(11) $$\begin{array}{c} 3\times \left(\text{A}+\text{B}\to \text{D}+2\text{HBr}\right)\\ 2\times \left(\text{A}+3\text{HBr}\to \text{A}{\left(\text{HBr}\right)}_{3}\right)\\ 5\text{A}+3\text{B}\to 3\text{D}+2\text{A}{\left(\text{HBr}\right)}_{3}. \end{array}$$

**Figure 3:**
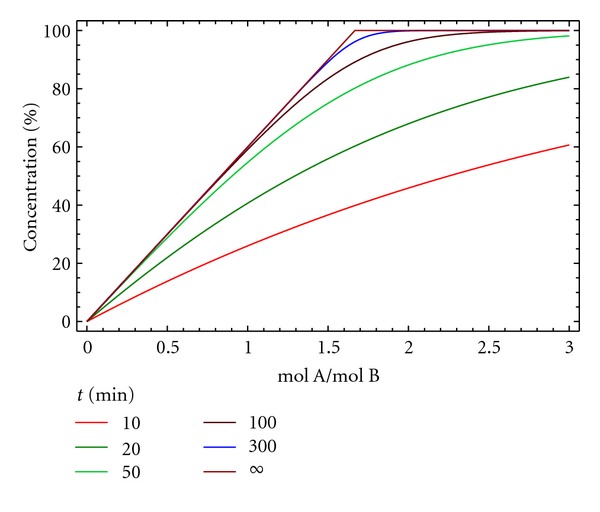
Yield of macrocycle D versus time for different initial molar ratios A/B at different reaction times for kinetic model 2. [A]_0_ = [B]_0_ = 0.005 M, *k*_1_ = 10 M^−1^ min^−1^ and *k*_2_ = 0.05 min^−1^.

Therefore, for being able to obtain a 100% yield, a 5 : 3 A/B initial stoichiometry is needed. The excess of A acts as a base neutralizing the hydrobromic acid formed, Figure 3.

### 2.3. Kinetic Model 3

**Scheme 4:**
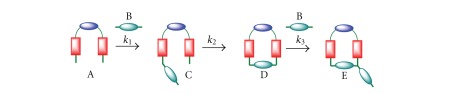
Reaction of macrocycle D with dihalide B.

 Once the macrocycle is formed according to kinetic model 1, a possible side reaction is its further reaction with dibromine B to yield product E (Scheme 4).

 Assuming that all reactions are irreversible, this can be represented according to (12)

(12) $$\begin{array}{c} \text{A}+\text{B}\overset{k_{1}}{\to }\text{C},\\ \text{C}\overset{k_{2}}{\to }\text{D},\\ \text{D}+\text{B}\overset{k_{3}}{\to }\text{E}. \end{array}$$

The starting materials A and B disappear by a second-order rate reaction

(13) $$\begin{array}{c} \frac{d\left[\text{A}\right]}{dt}=-k_{1}\left[\text{A}\right]\left[\text{B}\right]\\ \frac{d\left[\text{B}\right]}{dt}=-k_{1}\left[\text{A}\right]\left[\text{B}\right]-k_{3}\left[\text{D}\right]\left[\text{B}\right]. \end{array}$$

The reaction intermediate is formed by second-order rate reaction and disappears by a first-order rate reaction

(14) $$\frac{d\left[\text{C}\right]}{dt}=k_{1}\left[\text{A}\right]\left[\text{B}\right]-k_{2}\left[\text{C}\right].$$

The macrocyclic product D is formed by a first-order rate reaction and is consumed by a second-order reaction

(15) $$\frac{d\left[\text{D}\right]}{dt}=k_{2}\left[\text{C}\right]-k_{3}\left[\text{D}\right]\left[\text{B}\right].$$

The by-product E is formed by a second-order reaction, (16)

(16) $$\frac{d\left[\text{E}\right]}{dt}=k_{3}\left[\text{D}\right]\left[\text{B}\right].$$

**Figure 4:**
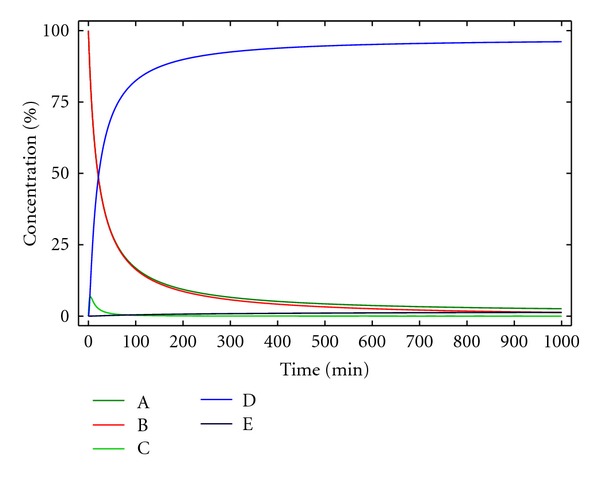
Concentration of the reactants, products, and reaction intermediates versus time for kinetic model 3. [A]_0_ = [B]_0_ = 0.005 M, *k*_1_ = 10 M^−1^ min^−1^, *k*_2_ = 0.5 min^−1^, and *k*_3_ = 0.05 M^−1^ min^−1^.

The solution of the differential equations (13)–(16) can be obtained numerically, as shown in Figure 4.

According to data in Figure 4 the percentage of the side product E increases with time and produces a decrease in the yield of the desired compound D that is twice the concentration of E, for a 1 : 1 A/B stoichiometry, as B has now two mechanisms for consumption. Thus, an increase in the initial concentration of A or a reduction in the reaction time is favourable for reducing the amount of E formed. However, although compound E has been identified as a side product in the studied reactions, it is always a minor by-product of the reaction. Thus, this kinetic model cannot be properly fitted to the experimental results.

### 2.4. Kinetic Model 4

**Scheme 5:**
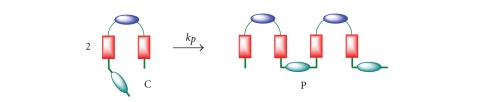
Reaction scheme for kinetic model 4. Reaction of C with C to give P.

**Scheme 6:**
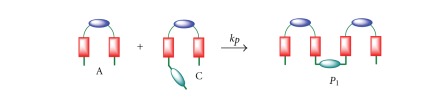
Reaction scheme for kinetic model 4. Reaction of C with A to give P_1_.

**Scheme 7:**
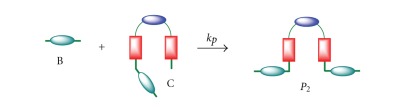
Reaction scheme for kinetic model 4. Reaction of C with B to give P_2_.

 This kinetic model takes into account the formation of oligomers/polymers besides the macrocyclization reaction. The reaction intermediate C can now react with A, B, or C, and the resulting oligomeric product (P, P_1_ or P_2_, see Schemes 5–7 and (17)), can further react with A, B, or C to produce higher-order oligomers. All those initial oligomeric products have been identified in the synthesis of macrocycle 2.

 Reaction of two molecules of C provides an oligomer P with one bromine at one end and one primary amine at the other end (Scheme 5). Reaction of C with A produces oligomer P_1_ with two primary amine groups at the ends (Scheme 6), while reaction with B gives the oligomer P_2_ with two bromine at the ends (Scheme 7). These oligomers can further react with A, B, C, P, and P_1_ y P_2_ to give larger oligomers and, eventually, polymers. As a result of the polymerization reactions, the yield of the macrocycle is reduced. These reactions are summarized in (17)

(17) $$\begin{array}{cc} \text{C}+\text{C}\overset{k_{p}}{\to }\text{P}\left(\text{Br},\text{N}\right) & \;\;{\text{P}}_{1}+\text{B}\overset{k_{p}}{\to }\text{P}\left(\text{Br},\text{N}\right)\\ \text{C}+\text{A}\overset{k_{p}}{\to }{\text{P}}_{1}\left(2\text{N}\right) & \;\;{\text{P}}_{1}+\text{C}\overset{k_{p}}{\to }{\text{P}}_{1}\left(2\text{N}\right)\\ \text{C}+\text{B}\overset{k_{p}}{\to }{\text{P}}_{2}\left(2\text{Br}\right) & \;\;{\text{P}}_{1}+\text{P}\overset{k_{p}}{\to }{\text{P}}_{1}\left(2\text{N}\right)\\ \text{A}+\text{B}\overset{k_{1}}{\to }\text{C} & \;\;{\text{P}}_{1}+{\text{P}}_{2}\overset{k_{p}}{\to }\text{P}\left(\text{Br},\text{N}\right)\\ \text{C}\overset{k_{2}}{\to }\text{D} & \;\;{\text{P}}_{2}+\text{A}\overset{k_{p}}{\to }\text{P}\left(\text{Br},\text{N}\right)\\ \text{P}+\text{A}\overset{k_{p}}{\to }{\text{P}}_{1}\left(2\text{N}\right) & \;\;{\text{P}}_{2}+\text{C}\overset{k_{p}}{\to }{\text{P}}_{2}\left(2\text{Br}\right)\\ \text{P}+\text{B}\overset{k_{p}}{\to }{\text{P}}_{2}\left(2\text{Br}\right) & \;\;{\text{P}}_{2}+\text{P}\overset{k_{p}}{\to }{\text{P}}_{2}\left(2\text{Br}\right).\\ \text{P}+\text{C}\overset{k_{p}}{\to }\text{P}\left(\text{Br},\text{N}\right) & \\ \text{P}+\text{P}\overset{k_{p}}{\to }\text{P}\left(\text{Br},\text{N}\right) & \end{array}$$

As mentioned above, we can assume that the kinetic rate constant of the polymerization steps is the same as the first reaction step (*k*_*p*_ = *k*_1_) as long as both nucleophilic substitution reactions yield open-chain compounds, and the strain involved for the macrocyclization step is not present. This assumption provides a simple kinetic model with only two constants (*k*_1_ and *k*_2_).

Thus, the starting materials disappear by second-order rate reactions

(18) $$\begin{array}{c} \frac{d\left[\text{A}\right]}{dt}=-k_{1}\left[\text{A}\right]\left[\text{B}\right]-k_{p}\left[\text{C}\right]\left[\text{A}\right]-k_{p}\left[\text{P}\right]\left[\text{A}\right]-k_{p}\left[{\text{P}}_{2}\right]\left[\text{A}\right]\\ \frac{d\left[\text{B}\right]}{dt}=-k_{1}\left[\text{A}\right]\left[\text{B}\right]-k_{p}\left[\text{C}\right]\left[\text{B}\right]-k_{p}\left[\text{P}\right]\left[\text{B}\right]-k_{p}\left[{\text{P}}_{1}\right]\left[\text{B}\right]. \end{array}$$

The reaction intermediate is formed by a second-order rate reaction and disappears by a first-order rate reaction and several second-order reactions yielding oligomeric/polymeric products

(19) $$\begin{array}{cc} & \frac{d\left[\text{C}\right]}{dt}=k_{1}\left[\text{A}\right]\left[\text{B}\right]-k_{2}\left[\text{C}\right]\\ & \;\;\;\;-k_{p}\left[\text{C}\right]\left[\text{A}\right]-k_{p}\left[\text{C}\right]\left[\text{B}\right]-2k_{p}\left[\text{C}\right]\left[\text{C}\right]\\ & \;\;\;\;-k_{p}\left[\text{C}\right]\left[\text{P}\right]-k_{p}\left[\text{C}\right]\left[{\text{P}}_{1}\right]-k_{p}\left[\text{C}\right]\left[{\text{P}}_{2}\right]. \end{array}$$

The macrocyclic product D is formed by a first-order rate reaction from the intermediate

(20) $$\frac{d\left[\text{D}\right]}{dt}=k_{2}\left[\text{C}\right].$$

**Figure 5:**
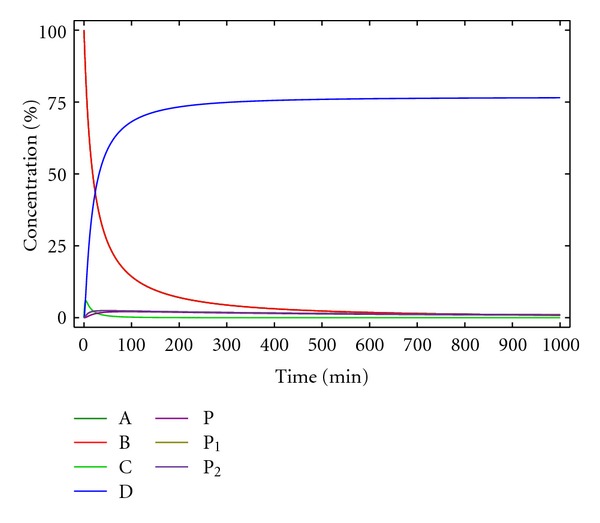
Concentration of the reactants, products, and reaction intermediates versus time for kinetic model 4. [A]_0_ = [B]_0_ = 0.005 M, *k*_1_ = 10 M^−1^ min^−1^, *k*_2_ = 0.5 min^−1^.

The polymeric products P, P_1_, and P_2_ are formed according to (21)–(23).

(21) $$\begin{array}{cc} & \frac{d\left[\text{P}\right]}{dt}=k_{p}\left[\text{C}\right]\left[\text{C}\right]-k_{p}\left[\text{P}\right]\left[\text{P}\right]\\ & \;\;\;\;-k_{p}\left[\text{P}\right]\left[\text{A}\right]-k_{p}\left[\text{P}\right]\left[\text{B}\right]+k_{p}\left[{\text{P}}_{1}\right]\left[\text{B}\right]\\ & \;\;\;\;-k_{p}\left[{\text{P}}_{1}\right]\left[\text{P}\right]+k_{p}\left[{\text{P}}_{1}\right]\left[{\text{P}}_{2}\right]\\ & \;\;\;\;+k_{p}\left[{\text{P}}_{2}\right]\left[\text{A}\right]-k_{p}\left[{\text{P}}_{2}\right]\left[\text{P}\right] \end{array}$$

(22) $$\frac{d\left[{\text{P}}_{1}\right]}{dt}=k_{p}\left[\text{C}\right]\left[\text{A}\right]+k_{p}\left[\text{P}\right]\left[\text{A}\right]-k_{p}\left[{\text{P}}_{1}\right]\left[\text{B}\right]-k_{p}\left[{\text{P}}_{1}\right]\left[{\text{P}}_{2}\right]$$

(23) $$\frac{d\left[{\text{P}}_{2}\right]}{dt}=k_{p}\left[\text{C}\right]\left[\text{B}\right]+k_{p}\left[\text{P}\right]\left[\text{B}\right]-k_{p}\left[{\text{P}}_{2}\right]\left[\text{A}\right]-k_{p}\left[{\text{P}}_{1}\right]\left[{\text{P}}_{2}\right],$$

where [A], [B], [C], [D], [P], [P_1_], and [P_2_] are the molar concentrations at time *t* of A, B, C, D, P, P_1_, and P_2_ respectively. The solution of differential equations (18)–(23) can again be obtained numerically as illustrated in Figure 5.

**Figure 6:**
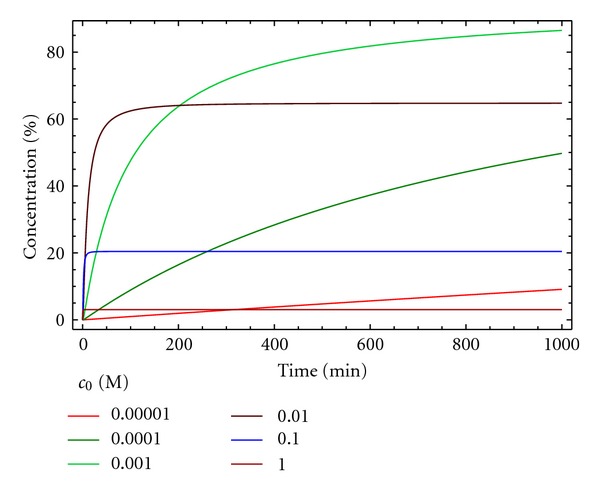
Concentration of the macrocycle D versus time for different initial concentrations ([A]_0_ = [B]_0_ = *c*_0_) for kinetic model 4. *k*_1_ = 10 M^−1^ min^−1^, *k*_2_ = 0.5 min^−1^.

For the conditions of Figure 5, at infinite time, the yield of D is not quantitative (75.9%), due to the oligomerization/polymerization reactions. Indeed, knowing the kinetic constants *k*_1_ and *k*_2_, the initial concentrations of A and B can be optimized to reduce the polymerization and perform the reaction in a reasonable reaction time. This can be achieved by plotting the concentration of the macrocycle D obtained for each initial concentration of reactants A and B (defined as *c*_0_ in Figure 6 for a 1 : 1 stoichiometry). As can be observed, the yield for D can be increased using lower concentrations of reactants, but if the dilution is too high, the reaction rate is too low and, accordingly, not practical. In the case described in Figure 6, best conditions are found for an initial concentration of reactants of 0.001 M, to obtain, in a reasonable time (400 min), a 80% yield.

**Figure 7:**
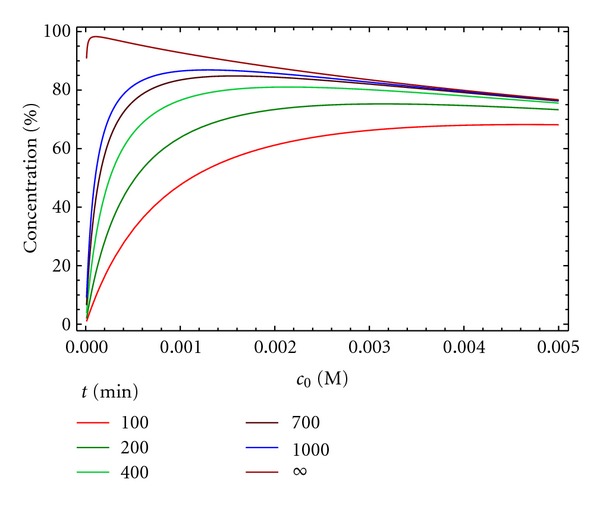
Concentration of the macrocycle D versus time at different reaction times ([A]_0_ = [B]_0_ = *c*_0_) for kinetic model 4. *k*_1_ = 10 M^−1^ min^−1^, *k*_2_ = 0.5 min^−1^.

An alternative representation involves plotting the concentration of the macrocycle D for each value of *c*_0_ at different reaction times (Figure 7). It can be observed that the yield, as expected, is 100% at infinite time for an initial concentration of reactants close to 0. As stated above, the use of a value for *c*_0_ of A and B around 0.001 M provides an optimal balance of time and yield. As oligomers are the main side products observed for these macrocyclization reactions, this kinetic model is more appropriate than model 3 for describing the experimental process. 

### 2.5. Kinetic Model 5

**Figure 8:**
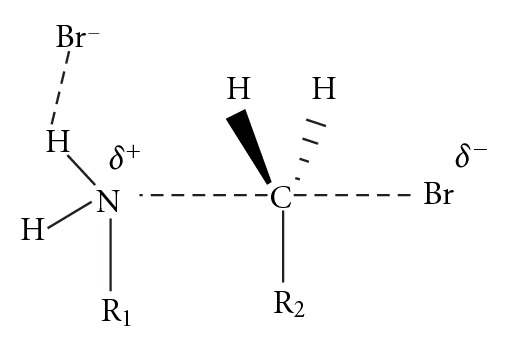
Transition state for the S_N_2 reaction stabilized by bromide anion.

 This mechanism assumes that the HBr formed in the S_N_2 reactions is neutralized by a base and the Br^−^ can act as a catalyst for the reaction. The anion can coordinate to the H atom of the amino group and stabilize the positive partial charge that is generated on the amino group as the nucleophilic substitution takes place, Figure 8. Therefore, the anion decreases the S_N_2 energy barrier that connects reactants and products acting as catalyst. The presence of a catalytic effect for different anions including Br^−^ has been observed in our group for different macrocyclization processes [8].

Therefore the chemical reactions that take place are displayed in (24).

(24) $$\begin{array}{c} \text{A}+\text{B}+\text{B}{\text{r}}^{-}\overset{k_{1c}}{\to }\text{C}+2\text{B}{\text{r}}^{-}\\ \text{C}+\text{B}{\text{r}}^{-}\overset{k_{2c}}{\to }\text{D}+\text{B}{\text{r}}^{-}. \end{array}$$

The starting materials disappear by a third-order rate reaction

(25) $$\frac{d\left[\text{A}\right]}{dt}=-k_{1c}\left[\text{A}\right]\left[\text{B}\right]\left[\text{B}{\text{r}}^{-}\right]$$

(26) $$\frac{d\left[\text{B}\right]}{dt}=-k_{1c}\left[\text{A}\right]\left[\text{B}\right]\left[\text{B}{\text{r}}^{-}\right].$$

The reaction intermediate is formed by a third-order rate reaction and disappears by a second-order rate reaction

(27) $$\frac{d\left[\text{C}\right]}{dt}=k_{1c}\left[\text{A}\right]\left[\text{B}\right]\left[\text{B}{\text{r}}^{-}\right]-k_{2c}\left[\text{C}\right]\left[\text{B}{\text{r}}^{-}\right].$$

Finally, the macrocyclic product D is formed by a first-order rate reaction

(28) $$\frac{d\left[\text{D}\right]}{dt}=k_{2c}\left[\text{C}\right]\left[\text{B}{\text{r}}^{-}\right].$$

The concentration of bromide increases with the progress of the reaction

(29) $$\frac{d\left[\text{B}{\text{r}}^{-}\right]}{dt}=k_{1c}\left[\text{A}\right]\left[\text{B}\right]\left[\text{B}{\text{r}}^{-}\right]+k_{2c}\left[\text{C}\right]\left[\text{B}{\text{r}}^{-}\right].$$

**Figure 9:**
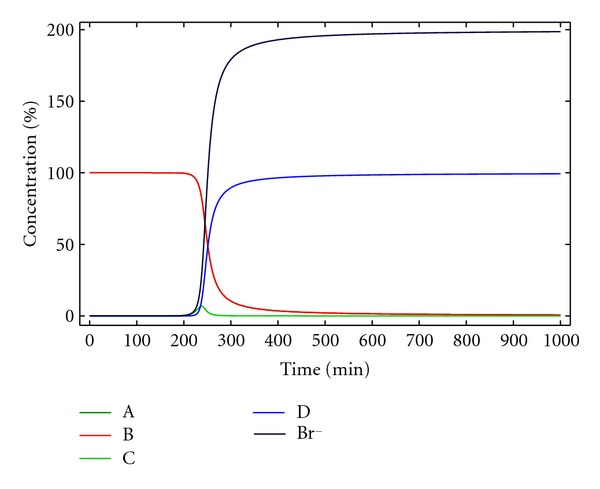
Concentration of the reactants, products, and reaction intermediates versus time for kinetic model 5. [A]_0_ = [B]_0_ = 0.005 M, [Br^−^]_0_ = 10^−12^ M, *k*_1*c*_ = 4000 M^−1^ min^−1^ and *k*_2*c*_ = 200 M^−1^ min^−1^.

This is an autocatalytic mechanism. If no Br^−^ is initially present, that is, [Br^−^]_0_ = 0, the reaction does not take place as long as [*A*]_0_ · [*B*]_0_ · [Br^−^]_0_ = 0. But if a small concentration Br^−^ is present at the beginning of the reaction, the reaction can proceed, and, after an induction time, the reaction starts to be accelerated as the concentration of bromide continuously increases (Figure 9).

**Figure 10:**
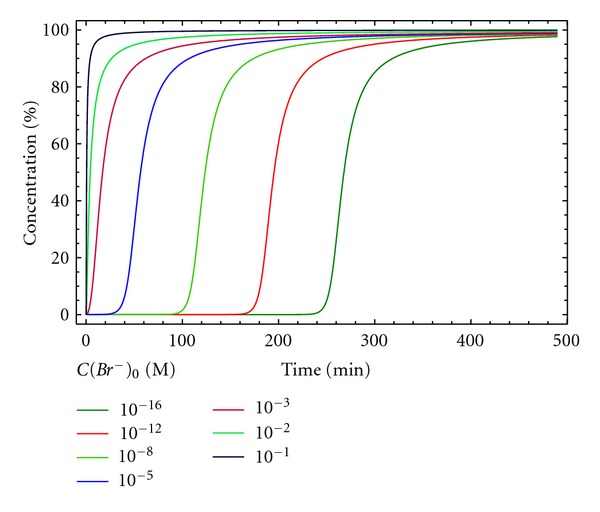
Concentration of the macrocycle D versus time, for different initial concentrations of Br^−^ for kinetic model 5. [A]_0_ = [B]_0_ = 0.005 M, *k*_1*c*_ = 4000 M^−2^ min^−1^ and *k*_2*c*_ = 200 M^−1^ min^−1^.

In this regard, Figure 10 shows how the induction period in the curve representing the concentration of macrocycle D, for the same initial concentration of A and B, is significantly reduced as the initial concentration of Br^−^ increases, providing a simple parameter for optimization. The lack of consideration of side products also limits the use of this kinetic model for a full description of the macrocyclizations considered in our work.

### 2.6. Kinetic Model 6

 For a better description of the experimental results observed in the synthesis of the pseudopeptidic macrocycles prepared in our work, the combination of some of the former models is necessary. Kinetic model 6 is a mixture of mechanisms 1 and 5, considering that the catalysed and the uncatalysed reactions can take place at the same time

(30) $$\;\begin{array}{c} \text{A}+\text{B}\overset{k_{1}}{\to }\text{C}+\text{B}{\text{r}}^{-}\\ \text{A}+\text{B}+\text{B}{\text{r}}^{-}\overset{k_{1c}}{\to }\text{C}+2\text{B}{\text{r}}^{-}\\ \text{C}\overset{k_{2}}{\to }\text{D}+\text{B}{\text{r}}^{-}\\ \text{C}+\text{B}{\text{r}}^{-}\overset{k_{2c}}{\to }\text{D}+\text{B}{\text{r}}^{-}. \end{array}$$

The starting materials A and B disappear by a third-order rate reaction

(31) $$\frac{d\left[\text{A}\right]}{dt}=-k_{1}\left[\text{A}\right]\left[\text{B}\right]-k_{1c}\left[\text{A}\right]\left[\text{B}\right]\left[\text{B}{\text{r}}^{-}\right]$$

(32) $$\frac{d\left[\text{B}\right]}{dt}=-k_{1}\left[\text{A}\right]\left[\text{B}\right]-k_{1c}\left[\text{A}\right]\left[\text{B}\right]\left[\text{B}{\text{r}}^{-}\right].$$

The evolution of the reaction intermediate follows (33).

(33) $$\frac{d\left[\text{C}\right]}{dt}=k_{1}\left[\text{A}\right]\left[\text{B}\right]-k_{2}\left[\text{C}\right]+k_{1c}\left[\text{A}\right]\left[\text{B}\right]\left[\text{B}{\text{r}}^{-}\right]-k_{2c}\left[\text{C}\right]\left[\text{B}{\text{r}}^{-}\right].$$

And the macrocyclic product D

(34) $$\begin{array}{cc} & \frac{d\left[\text{D}\right]}{dt}=k_{2}\left[\text{C}\right]+k_{2c}\left[\text{C}\right],\left[\text{B}{\text{r}}^{-}\right]\\ & \frac{d\left[\text{B}{\text{r}}^{-}\right]}{dt}=k_{1}\left[\text{A}\right]\left[\text{B}\right]+k_{2}\left[\text{C}\right]\\ & \;\;\;\;\;+k_{1c}\left[\text{A}\right]\left[\text{B}\right]\left[\text{B}{\text{r}}^{-}\right]+k_{2c}\left[\text{C}\right]\left[\text{B}{\text{r}}^{-}\right]. \end{array}$$

**Figure 11:**
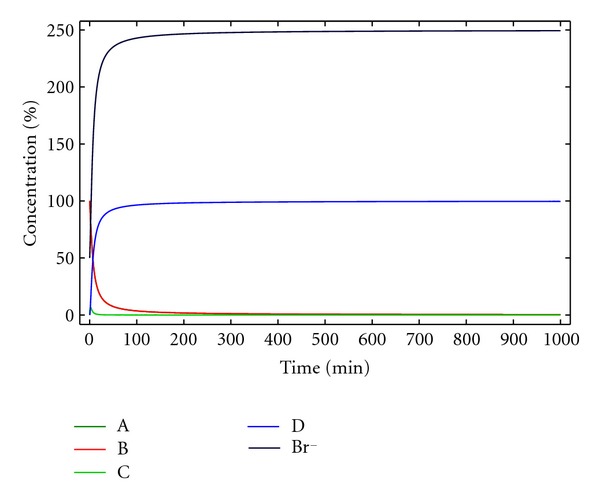
Concentration of the reactants, products, and reaction intermediates versus time for kinetic model 6. [A]_0_ = [B]_0_ = 0.005 M, [Br^−^]_0_ = 0.0025 M, *k*_1_ = 10 M^−1^ min^−1^, *k*_1*c*_ = 4000 M^−2^ min^−1^, *k*_2_ = 0.5 min^−1^, and *k*_2*c*_ = 200 M^−1^ min^−1^.

The numerical solution for this system is exemplified in Figure 11. This represents an advanced model relative to models 1 and 5 but still lacks the consideration of the formation of side products.

### 2.7. Kinetic Model 7

 This kinetic model is based on model 2 and 4. Thus, the formation of the main side products, oligomers/polymers, is introduced, but in the absence of base.

(35) $$\begin{array}{cc} \text{C}+\text{C}\overset{k_{p}}{\to }\text{P}\left(\text{Br},\text{N}\right)+\text{HBr} & \;\;{\text{P}}_{1}+\text{B}\overset{k_{p}}{\to }\text{P}\left(\text{Br},\text{N}\right)+\text{HBr}\\ \text{C}+\text{A}\overset{k_{p}}{\to }{\text{P}}_{1}\left(2\text{N}\right)+\text{HBr} & \;\;{\text{P}}_{1}+\text{C}\overset{k_{p}}{\to }{\text{P}}_{1}\left(2\text{N}\right)+\text{HBr}\\ \text{C}+\text{B}\overset{k_{p}}{\to }{\text{P}}_{2}\left(2\text{Br}\right)+\text{HBr} & \;\;{\text{P}}_{1}+\text{P}\overset{k_{p}}{\to }{\text{P}}_{1}\left(2\text{N}\right)+\text{HBr}\\ \text{A}+\text{B}\overset{k_{1}}{\to }\text{C}+\text{HBr} & \;\;{\text{P}}_{1}+{\text{P}}_{2}\overset{k_{p}}{\to }\text{P}\left(\text{Br},\text{N}\right)+\text{HBr}\\ \text{C}\overset{k_{2}}{\to }\text{D}+\text{HBr} & \;\;{\text{P}}_{2}+\text{A}\overset{k_{p}}{\to }\text{P}\left(\text{Br},\text{N}\right)+\text{HBr}\\ \text{A}+3\text{HBr}\to \text{A}{\text{H}}_{3}\text{B}{\text{r}}_{3}\left(\text{s}\right) & \;\;{\text{P}}_{2}+\text{C}\overset{k_{p}}{\to }{\text{P}}_{2}\left(2\text{Br}\right)+\text{HBr}\\ \text{P}+\text{A}\overset{k_{p}}{\to }{\text{P}}_{1}\left(2\text{N}\right)+\text{HBr} & \;\;{\text{P}}_{2}+\text{P}\overset{k_{p}}{\to }{\text{P}}_{2}\left(2\text{Br}\right)+\text{HBr}.\\ \text{P}+\text{B}\overset{k_{p}}{\to }{\text{P}}_{2}\left(2\text{Br}\right)+\text{HBr} & \\ \text{P}+\text{C}\overset{k_{p}}{\to }\text{P}\left(\text{Br},\text{N}\right)+\text{HBr} & \\ \text{P}+\text{P}\overset{k_{p}}{\to }\text{P}\left(\text{Br},\text{N}\right)+\text{HBr} & \end{array}$$

The starting materials A and B disappear by a second-order rate reaction

(36) $$\begin{array}{cc} & \frac{d\left[\text{A}\right]}{dt}=-k_{1}\left[\text{A}\right]\left[\text{B}\right]-k_{p}\left[\text{C}\right]\left[\text{A}\right]-k_{p}\left[\text{P}\right]\left[\text{A}\right]\\ & \;\;\;\;-k_{p}\left[{\text{P}}_{2}\right]\left[\text{A}\right]-\frac{1}{3}\frac{d\left[\text{HBr}\right]}{dt} \end{array}$$

(37) $$\frac{d\left[\text{B}\right]}{dt}=-k_{1}\left[\text{A}\right]\left[\text{B}\right]-k_{p}\left[\text{C}\right]\left[\text{B}\right]-k_{p}\left[\text{P}\right]\left[\text{B}\right]-k_{p}\left[{\text{P}}_{1}\right]\left[\text{B}\right].$$

The reaction intermediate is formed by a second-order rate reaction and disappears by a first-order rate reaction and second-order rate reactions yielding oligomeric products, (38).

(38) $$\begin{array}{cc} & \frac{d\left[\text{C}\right]}{dt}=k_{1}\left[\text{A}\right]\left[\text{B}\right]-k_{2}\left[\text{C}\right]-k_{p}\left[\text{C}\right]\left[\text{A}\right]-k_{p}\left[\text{C}\right]\left[\text{B}\right]\\ & \;\;\;\;-2k_{p}\left[\text{C}\right]\left[\text{C}\right]-k_{p}\left[\text{C}\right]\left[\text{P}\right]-k_{p}\left[\text{C}\right]\left[{\text{P}}_{1}\right]-k_{p}\left[\text{C}\right]\left[{\text{P}}_{2}\right]. \end{array}$$

The macrocyclic product D is formed by a first-order rate reaction

(39) $$\frac{d\left[\text{D}\right]}{dt}=k_{2}\left[\text{C}\right].$$

The polymeric products P, P_1_ and P_2_ are formed by second-order rate reactions in (40)–(42)

(40) $$\begin{array}{cc} & \frac{d\left[\text{P}\right]}{dt}=k_{p}\left[\text{C}\right]\left[\text{C}\right]-k_{p}\left[\text{P}\right]\left[\text{P}\right]-k_{p}\left[\text{P}\right]\left[\text{A}\right]\\ & \;\;\;\;-k_{p}\left[\text{P}\right]\left[\text{B}\right]+k_{p}\left[{\text{P}}_{1}\right]\left[\text{B}\right]-k_{p}\left[{\text{P}}_{1}\right]\left[\text{P}\right]\\ & \;\;\;\;+k_{p}\left[{\text{P}}_{1}\right]\left[{\text{P}}_{2}\right]+k_{p}\left[{\text{P}}_{2}\right]\left[\text{A}\right]-k_{p}\left[{\text{P}}_{2}\right]\left[\text{P}\right] \end{array}$$

(41) $$\frac{d\left[{\text{P}}_{1}\right]}{dt}=k_{p}\left[\text{C}\right]\left[\text{A}\right]+k_{p}\left[\text{P}\right]\left[\text{A}\right]-k_{p}\left[{\text{P}}_{1}\right]\left[\text{B}\right]-k_{p}\left[{\text{P}}_{1}\right]\left[{\text{P}}_{2}\right]$$

(42) $$\frac{d\left[{\text{P}}_{2}\right]}{dt}=k_{p}\left[\text{C}\right]\left[\text{B}\right]+k_{p}\left[\text{P}\right]\left[\text{B}\right]-k_{p}\left[{\text{P}}_{2}\right]\left[\text{A}\right]-k_{p}\left[{\text{P}}_{1}\right]\left[{\text{P}}_{2}\right].$$

The concentration of HBr increases as long as it is formed in each of the elemental reactions considered and decreases according to the process shown in (43).

(43) $$\;\begin{array}{cc} & \frac{d\left[\text{HBr}\right]}{dt}=k_{1}\left[\text{A}\right]\left[\text{B}\right]+k_{2}\left[\text{C}\right]+k_{p}\left[\text{C}\right]\left[\text{A}\right]\\ & \;\;\;\;+k_{p}\left[\text{C}\right]\left[\text{B}\right]+k_{p}\left[\text{C}\right]\left[\text{C}\right]+k_{p}\left[\text{P}\right]\left[\text{P}\right]\\ & \;\;\;\;+k_{p}\left[\text{P}\right]\left[\text{A}\right]+k_{p}\left[\text{P}\right]\left[\text{B}\right]+k_{p}\left[{\text{P}}_{1}\right]\left[\text{B}\right]+k_{p}\left[{\text{P}}_{1}\right]\left[\text{P}\right]\\ & \;\;\;\;+k_{p}\left[{\text{P}}_{1}\right]\left[{\text{P}}_{2}\right]+k_{p}\left[{\text{P}}_{2}\right]\left[\text{A}\right]+k_{p}\left[{\text{P}}_{2}\right]\left[\text{P}\right]. \end{array}$$

As in the case of kinetic model 2, the fast and quantitative neutralization of HBr formed has not been considered in (43) as this does not affect to the overall kinetics.

**Figure 12:**
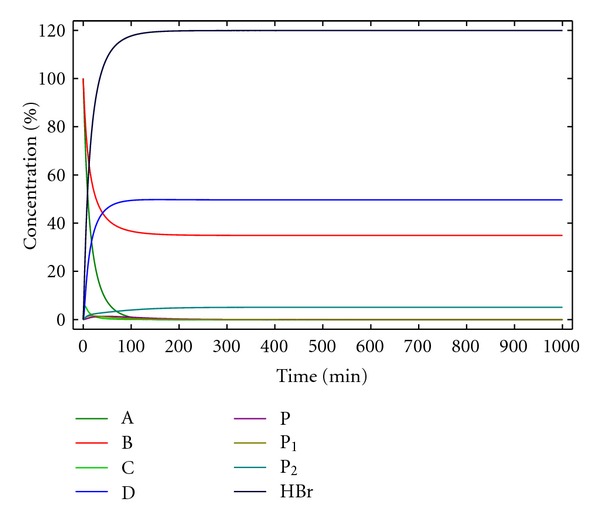
Concentration of the reactants, products, and reaction intermediates versus time for kinetic model 7. [A]_0_ = [B]_0_ = 0.005 M, [Br^−^]_0_ = 0.0025 M, *k*_1_ = 10 M^−1^ min^−1^, *k*_1*c*_ = 4000 M^−2^ min^−1^, *k*_2_ = 0.5 min^−1^, and *k*_2*c*_ = 200 M^−1^ min^−1^.

 The solution of this system of differential equations is outlined in Figure 12. Again, in this case, the maximum yield that can be obtained is relatively low, but as shown before, this can be solved through the use of an appropriate base. 

### 2.8. Kinetic Model 8

 This kinetic model takes the key idea of kinetic models 4, 5, and 6, that is, the uncatalysed and catalysed main reaction coexist, and the oligomerization/polymerization reactions are considered

(44) $$\begin{array}{cc} & \text{A}+\text{B}\overset{k_{1}}{\to }\text{C}+\text{B}{\text{r}}^{-}\\ & \text{A}+\text{B}+\text{B}{\text{r}}^{-}\overset{k_{1c}}{\to }\text{C}+2\text{B}{\text{r}}^{-}\\ & \text{C}\overset{k_{2}}{\to }\text{D}+\text{B}{\text{r}}^{-}\\ & \text{C}+\text{B}{\text{r}}^{-}\overset{k_{2c}}{\to }\text{D}+\text{B}{\text{r}}^{-}\\ & \text{C}+\text{C}\overset{k_{p}}{\to }\text{P}\left(\text{Br},\text{N}\right)+\text{B}{\text{r}}^{-}\\ & \text{C}+\text{A}\overset{k_{p}}{\to }{\text{P}}_{1}\left(2\text{N}\right)+\text{B}{\text{r}}^{-}\\ & \text{C}+\text{B}\overset{k_{p}}{\to }{\text{P}}_{2}\left(2\text{Br}\right)+\text{B}{\text{r}}^{-}\\ & \text{P}+\text{A}\overset{k_{p}}{\to }{\text{P}}_{1}\left(2\text{N}\right)+\text{B}{\text{r}}^{-}\\ & \text{P}+\text{B}\overset{k_{p}}{\to }{\text{P}}_{2}\left(2\text{Br}\right)+\text{B}{\text{r}}^{-}\\ & \text{P}+\text{C}\overset{k_{p}}{\to }\text{P}\left(\text{Br},\text{N}\right)+\text{B}{\text{r}}^{-}\\ & \text{P}+\text{P}\overset{k_{p}}{\to }\text{P}\left(\text{Br},\text{N}\right)+\text{B}{\text{r}}^{-}\\ & \text{C}+\text{C}+\text{B}{\text{r}}^{-}\overset{k_{pc}}{\to }\text{P}\left(\text{Br},\text{N}\right)+2\text{B}{\text{r}}^{-}\\ & \text{C}+\text{A}+\text{B}{\text{r}}^{-}\overset{k_{pc}}{\to }{\text{P}}_{1}\left(2\text{N}\right)+2\text{B}{\text{r}}^{-}\\ & \text{C}+\text{B}+\text{B}{\text{r}}^{-}\overset{k_{pc}}{\to }{\text{P}}_{2}\left(2\text{Br}\right)+2\text{B}{\text{r}}^{-}\\ & \text{P}+\text{A}+\text{B}{\text{r}}^{-}\overset{k_{pc}}{\to }{\text{P}}_{1}\left(2\text{N}\right)+2\text{B}{\text{r}}^{-}\\ & \text{P}+\text{B}+\text{B}{\text{r}}^{-}\overset{k_{pc}}{\to }{\text{P}}_{2}\left(2\text{Br}\right)+2\text{B}{\text{r}}^{-}\\ & \text{P}+\text{C}+\text{B}{\text{r}}^{-}\overset{k_{pc}}{\to }\text{P}\left(\text{Br},\text{N}\right)+2\text{B}{\text{r}}^{-}\\ & \text{P}+\text{P}+\text{B}{\text{r}}^{-}\overset{k_{pc}}{\to }\text{P}\left(\text{Br},\text{N}\right)+2\text{B}{\text{r}}^{-}\\ & {\text{P}}_{1}+\text{B}\overset{k_{p}}{\to }\text{P}\left(\text{Br},\text{N}\right)+\text{B}{\text{r}}^{-}\\ & {\text{P}}_{1}+\text{C}\overset{k_{p}}{\to }{\text{P}}_{1}\left(2\text{N}\right)+\text{B}{\text{r}}^{-}\\ & {\text{P}}_{1}+\text{P}\overset{k_{p}}{\to }{\text{P}}_{1}\left(2\text{N}\right)+\text{B}{\text{r}}^{-}\\ & {\text{P}}_{1}+{\text{P}}_{2}\overset{k_{p}}{\to }\text{P}\left(\text{Br},\text{N}\right)+\text{B}{\text{r}}^{-}\\ & {\text{P}}_{2}+\text{A}\overset{k_{p}}{\to }\text{P}\left(\text{Br},\text{N}\right)+\text{B}{\text{r}}^{-}\\ & {\text{P}}_{2}+\text{C}\overset{k_{p}}{\to }{\text{P}}_{2}\left(2\text{Br}\right)+\text{B}{\text{r}}^{-}\\ & {\text{P}}_{2}+\text{P}\overset{k_{p}}{\to }{\text{P}}_{2}\left(2\text{Br}\right)+\text{B}{\text{r}}^{-}\\ & {\text{P}}_{1}+\text{B}+\text{B}{\text{r}}^{-}\overset{k_{pc}}{\to }\text{P}\left(\text{Br},\text{N}\right)+2\text{B}{\text{r}}^{-}\\ & {\text{P}}_{1}+\text{C}+\text{B}{\text{r}}^{-}\overset{k_{pc}}{\to }{\text{P}}_{1}\left(2\text{N}\right)+2\text{B}{\text{r}}^{-}\\ & {\text{P}}_{1}+\text{P}+\text{B}{\text{r}}^{-}\overset{k_{pc}}{\to }{\text{P}}_{1}\left(2\text{N}\right)+2\text{B}{\text{r}}^{-}\\ & {\text{P}}_{1}+{\text{P}}_{2}+\text{B}{\text{r}}^{-}\overset{k_{pc}}{\to }\text{P}\left(\text{Br},\text{N}\right)+2\text{B}{\text{r}}^{-}\\ & \;{\text{P}}_{2}+\text{A}+\text{B}{\text{r}}^{-}\overset{k_{pc}}{\to }\text{P}\left(\text{Br},\text{N}\right)+2\text{B}{\text{r}}^{-}\\ & {\text{P}}_{2}+\text{C}+\text{B}{\text{r}}^{-}\overset{k_{pc}}{\to }{\text{P}}_{2}\left(2\text{Br}\right)+2\text{B}{\text{r}}^{-}\\ & {\text{P}}_{2}+\text{P}+\text{B}{\text{r}}^{-}\overset{k_{pc}}{\to }{\text{P}}_{2}\left(2\text{Br}\right)+2\text{B}{\text{r}}^{-} \end{array}$$

The starting materials A and B disappear according to (45) and (46)

(45) $$\begin{array}{cc} & \frac{d\left[\text{A}\right]}{dt}=-k_{1}\left[\text{A}\right]\left[\text{B}\right]-k_{p}\left[\text{C}\right]\left[\text{A}\right]\\ & \;\;\;-k_{p}\left[\text{P}\right]\left[\text{A}\right]-k_{p}\left[{\text{P}}_{2}\right]\left[\text{A}\right]-k_{1c}\left[\text{A}\right]\left[\text{B}\right]\left[\text{Br}\right]\\ & \;\;\;-k_{pc}\left[\text{C}\right]\left[\text{A}\right]\left[\text{Br}\right]-k_{pc}\left[\text{P}\right]\left[\text{A}\right]\left[\text{Br}\right]-k_{pc}\left[{\text{P}}_{2}\right]\left[\text{A}\right]\left[\text{Br}\right] \end{array}$$

(46) $$\begin{array}{cc} & \frac{d\left[\text{B}\right]}{dt}=-k_{1}\left[\text{A}\right]\left[\text{B}\right]-k_{p}\left[\text{C}\right]\left[\text{B}\right]\\ & \;\;\;-k_{p}\left[\text{P}\right]\left[\text{B}\right]-k_{p}\left[{\text{P}}_{1}\right]\left[\text{B}\right]-k_{1c}\left[\text{A}\right]\left[\text{B}\right]\left[\text{Br}\right]\\ & \;\;\;-k_{pc}\left[\text{C}\right]\left[\text{B}\right]\left[\text{Br}\right]-k_{pc}\left[\text{P}\right]\left[\text{B}\right]\left[\text{Br}\right]-k_{pc}\left[{\text{P}}_{1}\right]\left[\text{B}\right]\left[\text{Br}\right] \end{array}$$

The variation of the concentration of the reaction intermediate with the time is described by (47)

(47) $$\begin{array}{cc} \frac{d\left[\text{C}\right]}{dt} & =k_{1}\left[\text{A}\right]\left[\text{B}\right]-k_{2}\left[\text{C}\right]-k_{p}\left[\text{C}\right]\left[\text{A}\right]-k_{p}\left[\text{C}\right]\left[\text{B}\right]\\ & \;\;-2k_{p}\left[\text{C}\right]\left[\text{C}\right]-k_{p}\left[\text{C}\right]\left[\text{P}\right]-k_{p}\left[\text{C}\right]\left[{\text{P}}_{1}\right]\\ & \;\;-k_{p}\left[\text{C}\right]\left[{\text{P}}_{2}\right]+k_{1c}\left[\text{A}\right]\left[\text{B}\right]\left[\text{Br}\right]-k_{2c}\left[\text{C}\right]\left[\text{Br}\right]\\ & \;\;-k_{pc}\left[\text{C}\right]\left[\text{A}\right]\left[\text{Br}\right]-k_{pc}\left[\text{C}\right]\left[\text{B}\right]\left[\text{Br}\right]\\ & \;\;-2k_{pc}\left[\text{C}\right]\left[\text{C}\right]\left[\text{Br}\right]-k_{pc}\left[\text{C}\right]\left[\text{P}\right]\left[\text{Br}\right]\\ & \;\;-k_{p\text{c}}\left[\text{C}\right]\left[{\text{P}}_{1}\right]\left[\text{Br}\right]-k_{pc}\left[\text{C}\right]\left[{\text{P}}_{2}\right]\left[\text{Br}\right]. \end{array}$$

And the macrocyclic product D is formed by a first-order rate reaction for the uncatalysed macrocyclization reaction and by a second-order reaction for the catalysed reaction

(48) $$\frac{d\left[\text{D}\right]}{dt}=k_{2}\left[\text{C}\right]+k_{2c}\left[\text{C}\right]\left[\text{Br}\right].$$

The corresponding differential equations for the polymeric products P, P_1_, and P_2_ are described in (49)–(51)

(49) $$\begin{array}{cc} \frac{d\left[\text{P}\right]}{dt} & =k_{p}\left[\text{C}\right]\left[\text{C}\right]-k_{p}\left[\text{P}\right]\left[\text{P}\right]-k_{p}\left[\text{P}\right]\left[\text{A}\right]-k_{p}\left[\text{P}\right]\left[\text{B}\right]\\ & \;\;\;+k_{p}\left[{\text{P}}_{1}\right]\left[\text{B}\right]-k_{p}\left[{\text{P}}_{1}\right]\left[\text{P}\right]+k_{p}\left[{\text{P}}_{1}\right]\left[{\text{P}}_{2}\right]\\ & \;\;\;+k_{p}\left[{\text{P}}_{2}\right]\left[\text{A}\right]-k_{p}\left[{\text{P}}_{2}\right]\left[\text{P}\right]\\ & \;\;\;+k_{pc}\left[\text{C}\right]\left[\text{C}\right]\left[\text{Br}\right]-k_{pc}\left[\text{P}\right]\left[\text{P}\right]\left[\text{Br}\right]\\ & \;\;\;-k_{pc}\left[\text{P}\right]\left[\text{A}\right]\left[\text{Br}\right]-k_{pc}\left[\text{P}\right]\left[\text{B}\right]\left[\text{Br}\right]\\ & \;\;\;+k_{pc}\left[{\text{P}}_{1}\right]\left[\text{B}\right]\left[\text{Br}\right]-k_{pc}\left[{\text{P}}_{1}\right]\left[\text{P}\right]\left[\text{Br}\right]\\ & \;\;\;+k_{pc}\left[{\text{P}}_{1}\right]\left[{\text{P}}_{2}\right]\left[\text{Br}\right]\\ & \;\;\;+k_{pc}\left[{\text{P}}_{2}\right]\left[\text{A}\right]\left[\text{Br}\right]-k_{pc}\left[{\text{P}}_{2}\right]\left[\text{P}\right]\left[\text{Br}\right] \end{array}$$

(50) $$\begin{array}{cc} & \frac{d\left[{\text{P}}_{1}\right]}{dt}=k_{p}\left[\text{C}\right]\left[\text{A}\right]+k_{p}\left[\text{P}\right]\left[\text{A}\right]-k_{p}\left[{\text{P}}_{1}\right]\left[\text{B}\right]\\ & \;\;\;\;-k_{p}\left[{\text{P}}_{1}\right]\left[{\text{P}}_{2}\right]+k_{pc}\left[\text{C}\right]\left[\text{A}\right]\left[\text{Br}\right]\\ & \;\;\;\;+k_{pc}\left[\text{P}\right]\left[\text{A}\right]\left[\text{Br}\right]\\ & \;\;\;\;-k_{pc}\left[{\text{P}}_{1}\right]\left[\text{B}\right]\left[\text{Br}\right]-k_{pc}\left[{\text{P}}_{1}\right]\left[{\text{P}}_{2}\right]\left[\text{Br}\right] \end{array}$$

(51) $$\begin{array}{cc} & \frac{d\left[{\text{P}}_{2}\right]}{dt}=k_{p}\left[\text{C}\right]\left[\text{B}\right]+k_{p}\left[\text{P}\right]\left[\text{B}\right]-k_{p}\left[{\text{P}}_{2}\right]\left[\text{A}\right]\\ & \;\;\;\;-k_{p}\left[{\text{P}}_{1}\right]\left[{\text{P}}_{2}\right]+k_{pc}\left[\text{C}\right]\left[\text{B}\right]\left[\text{Br}\right]\\ & \;\;\;\;+k_{pc}\left[\text{P}\right]\left[\text{B}\right]\left[\text{Br}\right]\\ & \;\;\;\;-k_{pc}\left[{\text{P}}_{2}\right]\left[\text{A}\right]\left[\text{Br}\right]-k_{pc}\left[{\text{P}}_{1}\right]\left[{\text{P}}_{2}\right]\left[\text{Br}\right]. \end{array}$$

**Figure 13:**
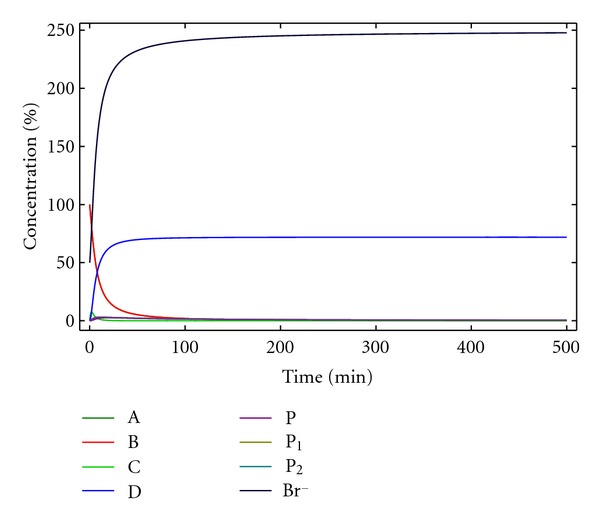
Concentration of the reactants, products, and reaction intermediates versus time for kinetic model 8. [A]_0_ = [B]_0_ = 0.005 M, [Br^−^]_0_ = 0.0025 M, *k*_1_ = 10 M^−1^ min^−1^, *k*_1*c*_ = 4000 M^−2^ min^−1^, *k*_2_ = 0.5 min^−1^, and *k*_2*c*_ = 200 M^−1^ min^−1^.

Although this is a complex system, the numerical solution of differential equations (45)–(51) are possible, as illustrated in Figure 13. On the other hand, this model considers the most important parameters found in the experimental process for the preparation of macrocycles such as 1 and 2, that is, the use of an appropriate base for neutralizing the HBr formed, the presence of oligomerization processes as the main side reactions, the catalytic effect of the Br^−^ anion, as well as the presence of some uncatalysed reaction. According to the experimental evidence, the formation of the side product considered in model 3 is very minor, and the corresponding equations have not been considered here.

**Figure 14:**
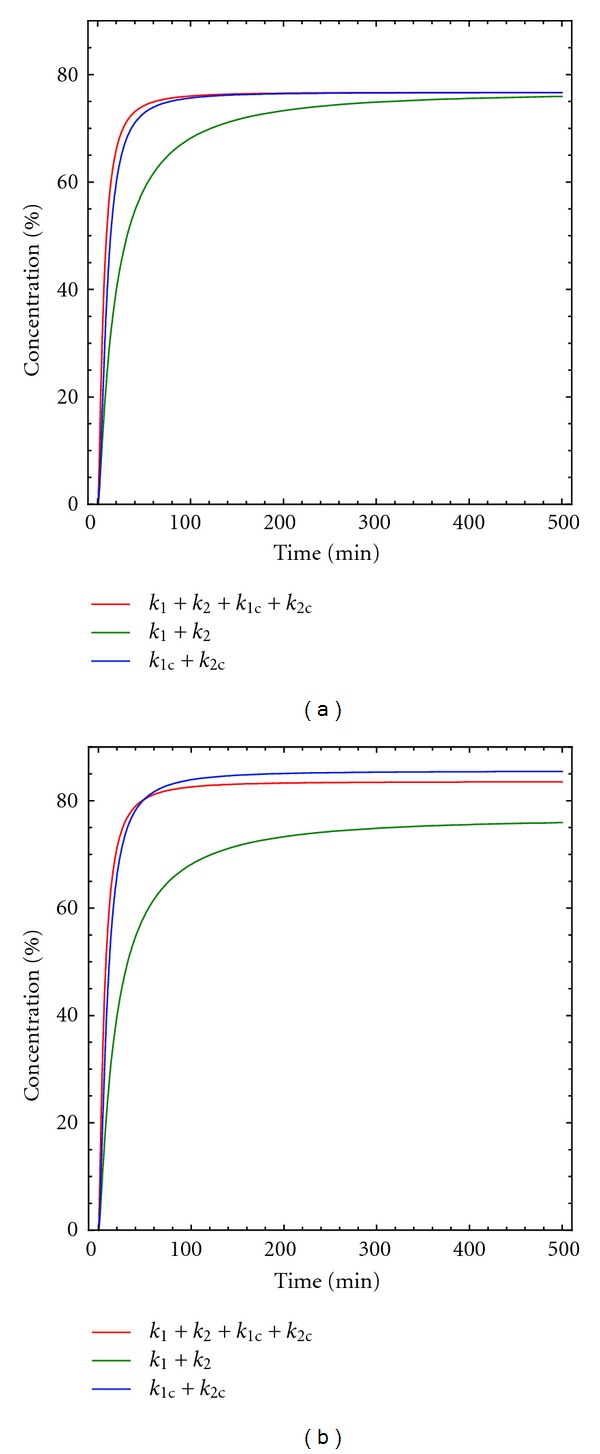
Concentration macrocycle D versus time for kinetic model 8. [A]_0_ = [B]_0_ = 0.005 M, [Br^−^]_0_ = 0.0025 M, *k*_1_ = 10 M^−1^ min^−1^, *k*_1*c*_ = 4000 M^−2^ min^−1^, *k*_2_ = 0.5 min^−1^, (a) *k*_2*c*_ = 200 M^−1^ min^−1^, and (b) *k*_2*c*_ = 400 M^−1^ min^−1^.

 Although this is a complicated model, it provides a simple analysis for understanding the regulation and optimization of the process. Thus, if the *k*_2_/*k*_1_, and *k*_2*c*_/*k*_1*c*_ ratios are identical or relatively similar (0.05 in Figure 14(a)), the final yields are essentially identical. However, if the *k*_2*c*_/*k*_1*c*_ ratio is bigger than the *k*_2_/*k*_1_ the yield for the macrocyclic product will be incremented (Figure 14(b)) according to the catalytic effect of the anion. These figures represent the variation with time of the concentration of the desired product for three different situations; only the non-catalysed reactions are involved (*k*_2_/*k*_1_), only the catalysed process is involved (*k*_2*c*_/*k*_1*c*_), and both mechanisms are participating (*k*_2_/*k*_1_ + *k*_2*c*_/*k*_1*c*_).

**Figure 15:**
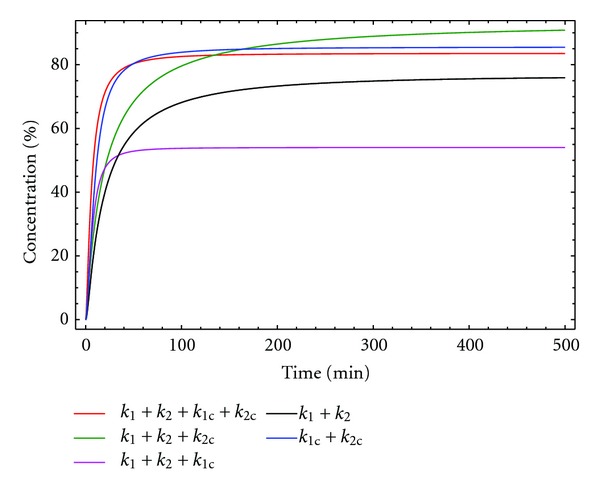
Concentration macrocycle D versus time for kinetic model 8. [A]_0_ = [B]_0_ = 0.005 M, [Br^−^]_0_ = 0.0025 M, *k*_1_ = 10 M^−1^  min^−1^, *k*_1*c*_ = 4000 M^−2^ min^−1^, *k*_2_ = 0.5 min^−1^ and *k*_2*c*_ = 400 M^−1^ min^−1^.

 As shown in Figure 15, the effect of the catalysis on both reaction steps is very different. The effect on the second reaction step is bigger than that observed for the first reaction step.

Thus, this kinetic model represents a good starting point for the analysis of the macrocyclization reactions under study and for obtaining the corresponding kinetic parameter from experimental data. Nevertheless, the presence of four different adjustable parameters (*k*_2_/*k*_1_ + *k*_2*c*_/*k*_1*c*_) can lead to misleading results. Fortunately, the results shown in Figures 14 and 15 clearly reveal that, for a true catalytic process, the contribution of the non-catalysed reactions is very minor and can be disregarded. In this case, only two adjustable parameters are present, and the fitting of the experimental data to the model is excellent, providing kinetic parameters that are also in good agreement with the trends obtained from theoretical calculations [8].

 An even more complex kinetic model can be elaborated by combining the kinetic model 8 with kinetic model 3. This involves considering the formation of the side product E along with that of the different oligomers/polymers. Introducing this additional reaction requires the presence of an additional adjustable parameter (*k*_3_) when the fitting of the experimental data to the model is attempted, which is again a factor that could be difficult obtaining data of physical relevance. However, according to experimental data showing that E is always a very minor side product, it must be considered that *k*_3_ ≪ *k*_1_. Using the same reasoning used above in the case of the catalysed/uncatalysed processes, it can be demonstrated that the influence of this side reaction on the overall process is minimal and can also be disregarded without significantly reducing the accuracy of the analysis and that of the numeric results obtained for the fitting of the experimental data to the model.

## 3. Conclusions

 We have developed a broad set of kinetic models for the macrocyclization reaction of bis(amidoamines) with dihalides, considering the different steps involved as well as the different potential alternative reactions leading to side products that have been observed experimentally. Such kinetic models vary from the most simple one, where only the two main reaction steps are considered, to the most complicated one, where polymerization reactions and the effect of catalytic anions have been included. The solution of all those kinetic models can be easily obtained numerically with the use of accessible tools such as Mathematica. The comprehensive analysis of the solutions of the kinetic models allows to predict the macrocyclization yield from a given set of kinetic constants and initial concentrations. Although the most adequate description of the process can be associated to the most complex model, the use of the simple models allows to easily understand the experimental parameters that permit implementing or reducing the corresponding process (main or side process). The analysis here presented can also be of interest for the study of other related macrocyclization processes as much as the main reactions and the side reactions considered have been shown to be similar in most of them. Moreover, fitting of the model to experimental kinetic data allows obtaining the experimental kinetic constants from kinetic experiments. However, when using the most complex model, the presence of an excessive number of adjustable parameters can lead to mathematical results lacking a true physical relevance. The analysis presented for this kinetic model indicates, however, that, in the presence of a significant catalytic pathway for the macrocyclization, the contribution of the noncatalysed macrocyclization is very much reduced. This allows for a further simplification, providing a model with only two kinetic adjustable parameters. This has been shown to provide an excellent fitting to experimental results and to obtain the corresponding experimental values for the kinetic constants of the two steps involved in the macrocyclization.
